# Neuroticism and Frontal EEG Asymmetry Correlated With Dynamic Facial Emotional Processing in Adolescents

**DOI:** 10.3389/fpsyg.2019.00175

**Published:** 2019-02-08

**Authors:** Seyedeh Maryam Moshirian Farahi, Mohammad Javad Asghari Ebrahimabad, Ali Gorji, Imanollah Bigdeli, Seyed Mohammad Mahdi Moshirian Farahi

**Affiliations:** ^1^Department of Psychology, Ferdowsi University of Mashhad, Mashhad, Iran; ^2^Department of Neurology, Epilepsy Research Center, University of Münster, Münster, Germany; ^3^Department of Neurosurgery, Epilepsy Research Center, University of Münster, Münster, Germany; ^4^Department of Psychology, Carleton University, Ottawa, ON, Canada

**Keywords:** frontal EEG asymmetry, dynamic facial emotional processing, the valence, neuroticism, psychopathology, fear

## Abstract

The aim of this research was to investigate the link between resting frontal EEG asymmetry, neuroticism and the valence of emotional face processing in adolescents. Fifty right-handed adolescents (50% male; mean age = 14.20, *SD* = 1.97) were selected from schools in Mashhad. In order to investigate variables, we used BFQ-C, ADFES-BIV, and EEG. All data were analyzed using SPSS 22. The results showed that neuroticism correlates with the valences of fear, disgust, sadness, and surprise, but not with happiness, anger, and neutral faces. Furthermore, it was found that N was significantly positively correlated with mid-frontal asymmetry (F3–F4), and the lateral-frontal (F7–F8), whereas no correlation was found between N and frontal pole (Fp1–Fp2). We found significant negative correlations between the valence of fear, Fp1–Fp2, F3–F4, and F7–F8. The interaction findings revealed that neuroticism^∗^mid-frontal asymmetry can significantly affect the valence of fear. Therefore, neuroticism and mid-frontal EEG asymmetry may serve as a risk indicator for psychopathology.

## Introduction

Similar adults, personality traits of children and adolescents can be described in terms of characteristic patterns of feeling, thinking, and behavior. There are both similarities and differences between adolescent and adult personality traits. Adolescents’ personality traits help to shape the course of their lives, but a full understanding of adolescents’ personality traits requires additional investigation on the intersection of personality as well as clinical and developmental psychology (Soto and Tackett, 2015). One such trait is neuroticism, which has been associated with an increased sensitivity to fear, anxiety, and distress (McCrae and Costa, 1999; DeYoung, 2015). According to the Eysenck model, individuals with a high-level of neuroticism is more responsible to the negative emotions related to the limbic system (Eysenck and Eysenck, 1985). Many researchers are interested in reviewing neuroticism as it may predict the inclination of the individual toward emotional and anxiety-related disorders (Kovalenko et al., 2010). Individuals with high levels of neuroticism are prone to emotional responses to stress that foster avoidant or defensive behaviors, including anxiety, depression, anger, irritability, and panic attacks, making it the major personality risk factor for the development of psychopathology (Lahey, 2009). People who are high in neuroticism are more probable to respond to emotional stimuli, and they have a low threshold for negative emotions (Minnix and Kline, 2004). Studies have shown a positive relationship between neuroticism and negative affect (Ng, 2009). One of the major emotional stimuli in social interactions is the human face. Paul Ekman (1993) originally posted hardwired programs that link the basic emotions (happiness, sadness, fear, disgust, anger, surprise, and contempt) to specific facial expressions. Emotions and moods contribute to a human’s decision-making process, which can result in either positive or negative consequences for subsequent behavior. Furthermore, face perception is treated as an advanced visual detection skill in humans (Theeuwes and Van der Stigchel, 2006). The results of empirical studies have evidenced that two personality traits, anxiety and aggression, are especially associated with biases involved in perceiving emotional events (Pishyar et al., 2004). Individuals in severe distress are more likely to reveal rumors regarding negative social stimuli, and people with great anxiety tend to respond to furious emotional expressions as threatening rather than neutral social stimuli (Mogg and Bradley, 2002).

The mechanisms underlying the interaction between traits and emotion are poorly understood. All persistent individual differences in motivation, cognition, thought, and emotion must require patterns of agreement in the functioning of the brain. From this perspective, the brain is the proximal source of all personality characteristics (DeYoung et al., 2010). Researchers have examined the relationship between these personality constructs, emotional processing and brain activity using psychophysiological measures, consisting of frontal asymmetry in the electroencephalogram (EEG) as indexed by the alpha band (Grimshaw and Carmel, 2014). Alpha oscillations (8–12 Hz) play a key role in awareness and attentional control (Hanslmayr et al., 2011; Mazaheri et al., 2013). Frontal asymmetric alpha levels are linked to greater cortical activity in the hemisphere with a lower alpha activity (Coan and Allen, 2004; Mathersul et al., 2008).

Four main frontal asymmetry models have proposed to describe frontal asymmetry and emotional processing, including motivational trait tendencies model (Davidson, 1998), and the valence-arousal model (Heller, 1993), the capability model (Coan et al., 2006), and the asymmetric inhibition model (Grimshaw and Carmel, 2014). According to the motivational trait tendencies model (Davidson, 1998; Harmon-Jones, 2003), EEG frontal asymmetry mediates and moderates motivational tendencies to approach/withdraw underlying emotions. Underlying the valence-arousal model, the valence of emotions are more important than motivational tendencies (Tomarken et al., 1992; Heller, 1993, 2013). Numerous studies have revealed that relatively higher left frontal activity is associated with a general appetitive, approach, or behavioral activation motivational system. In contrast, relatively higher right frontal activity is associated with the avoidance or withdrawal system (Coan and Allen, 2004). EEG studies have shown a pattern of higher activation in the right frontal lobe when observing stimuli or resting (Shackman et al., 2009). For example, greater relative left frontal activity might be expected to be associated with greater self-reported happiness, and greater relative right frontal activity might be predicted to response to the negative valence of stimuli. Tomarken et al. (1990) showed that resting alpha power of frontal asymmetry significantly predicts self-reported global negative affect in response to film clips and predict the difference between global positive and negative affect.

Though studies have indicated that the valence of negative emotions are related to the right frontal activity and the valence of positive emotions are linked to the left frontal activity, there are documents revealed that this relationship does not occur for anger faces. Accordingly, the valence of anger face is associated with the left frontal activity. In the literature, emotional states such as anger, joy, and surprise are indexed as approach-oriented emotional states (Harmon-Jones and Sigelman, 2001; Coan and Allen, 2004). Thus, instead of the valence-arousal model, it is concluded that frontal asymmetry reflects motivational tendencies rather than the valence model (Harmon-Jones, 2004; Hewig et al., 2004; Gable and Poole, 2014; Palmiero and Piccardi, 2017).

However, we believe that these models only provide a description of EEG frontal asymmetry of emotional processing, but not provide a specific mechanism of frontal asymmetry. After two primary models, Coan and Allen (2004) doubted the asymmetry model. According to Coan and Allen (2004), asymmetry can be classified into the following four categories: frontal EEG asymmetry is investigated as (1) an individual difference related to trait-like measures; (2) an individual difference that can anticipate state-related emotional changes and responses; (3) an individual difference linked to psychopathological risk factors, such as anxiety and depression; and (4) a function of state changes in emotion by exploring state-related change in asymmetry. The first three categories of studies obviously suppose that frontal asymmetry has trait-like properties, while the fourth assumes that state-related changes in EEG asymmetry can be evoked and observed. Resting frontal EEG asymmetry taps an individual difference that may simplify or dwindle an emotional response across many categories of stimuli (Coan and Allen, 2004). Coan and Allen (2004) proposed explaining frontal asymmetry as mediator or moderator variables to predict emotional responses.

In the third model, Coan et al. (2006) proposed the capability model. This model reveal that different patterns of EEG frontal activity are influenced by situations and contexts, reflecting a capability to adapt to the situation that rely on activating emotion regulation and inhibiting emotional responses when needed. The capability model focuses on emotional challenges during a task and the model believes that measuring frontal asymmetry during a task is more powerful and reliable than a resting state (Coan et al., 2006). Moody (2016) indicated that the capability model does not provide the specific mechanisms through which such emotional management might occur. However, it describes an important shift of focus toward cognitive explanations of frontal asymmetry (Moody, 2016).

Grimshaw and Carmel (2014) designed the asymmetric inhibition model (the fourth model) based on electro physiological, neurological, and clinical research. The model provides that frontal asymmetry reflects the cognitive control based on the dorso-lateral of the prefrontal cortex region (dlPFC). The dlPFC plays a key role in executive functions, including working memory, shifting, inhibition, updating (Miyake and Friedman, 2012), and attentional network (Vossel et al., 2014). The model suggests that left dlPFC is responsible for inhibition of negative distractors, while right dlPFC is responsible for inhibition of positive distractors. Lower activity of left frontal region reflects less effective inhibition of negative information that is related to depression and anxiety. Lower activity of right frontal region reflects less effective inhibition of positive information that is related to addiction and low level of self-regulation (Smit et al., 2007; Grimshaw and Carmel, 2014; Moody, 2016; Palmiero and Piccardi, 2017).

Several investigations evaluated the mutual relationship between neuroticism and frontal asymmetry. Using MRI, Forbes et al. (2014) identified a link between greater scores on neuroticism and focal damage to the left dorsolateral prefrontal cortex in patients with brain injury. There is a body of evidence that neuroticism is associated with lateralization, which is in turn triggered by variation in frontal lobe activation. More importantly, this association with hemispheric asymmetry is not demonstrated in all components of neuroticism. This right-dominant asymmetry seems to be applicable to traits in the withdrawal sub-factors, such as anxiety and depression, and is associated with passive avoidance. In contrast, traits of the volatility sub-factors, including anger-susceptibility and aggressiveness, which are involved in active defense, are linked to higher left frontal asymmetry (Allen and DeYoung, 2016).

Some studies on the normal population have not found a link between neuroticism and frontal asymmetry (Tomarken and Davidson, 1994; Kline et al., 2002), whereas Gale et al. (2001) showed that decreased activity in the right hemisphere is related to neuroticism. Moreover, Minnix and Kline (2004) revealed that increasing mid-frontal asymmetry variability is associated with higher levels of neuroticism. As regard to the Minnix and Kline’s (2004) findings, EEG asymmetry variability may be related to a tendency toward fluctuation between positive and negative emotional states. On the other hand, this variation may be related to slides between anxious arousal and anxious apprehension. Therefore, there are inconsistent findings in the relation between EEG frontal asymmetry and neuroticism.

As neuroticism trait trends to negative emotions, and negatively valences negative emotions related to the psychological problems such as depression, anxiety, impulsivity, and aggression; therefore, it is an important trait to predict psychopathological problems. Studies have shown that there are relationships between the brain frontal asymmetry in alpha wave, neuroticism, and emotions. However, these relationships did not examine neuroticism and frontal asymmetry interactions to predict valences of emotions. Accordingly, we intended to examine the relationship between neuroticism and valences of emotions by interacting frontal asymmetry. In fact, we firstly examined the relationship between behavioral data (neuroticism and emotional valences). We secondly examined correlations between valences of emotions, neuroticism as a trait, and frontal asymmetry to test the asymmetric inhibition model (Grimshaw and Carmel, 2014). We thirdly tested frontal asymmetry as a moderator variable according to proposal Coan and Allen’s (2004) in order to integrate interactions of individual differences according to neuroticism and frontal asymmetry to predict valences of emotions. The present study conducted in adolescents; because this developmental period is a critical stage in clarifying pathological aspects of the next stage of development. Furthermore, since the previous studies have revealed that dynamic facial expressions are recognized more accurately and faster than static faces (Calvo et al., 2016); thus, in this research, we applied dynamic emotional faces for valence ratings.

## Materials and Methods

### Participants

Fifty right-handed adolescents (50% male) were selected from schools in Mashhad. The age range of participants was from 11 to 18 years with a mean of 14.20 years (*SD* = 1.97). All participants gave informed consent, and all were free of specific psychological and neurological diseases.

### Questionnaires

#### The Big Five Questionnaire for Children (BFQ-C)

The BFQ-C (Barbaranelli and Caprara, 2000; Barbaranelli et al., 2003) is a 65-item questionnaire assessing the five factors of personality in children and adolescents. The first, Extraversion, measures characteristics such as enthusiasm, activity, self-confidence, and assertiveness. Agreeableness reflects sensitivity and concern for others and their needs, whereas Conscientiousness measures orderliness, the fulfilling of commitments, precision, and dependability. Neuroticism appertains to experience feelings of discontent, anxiety, anger, and depression. Finally, Openness is interested in intellectual functioning, imagination, creativity, and social and cultural interest. Rating of the questionnaire is according to a 5-point Likert scale ranging from 1 (almost never) to 5 (almost always). Individual item scores are combined to yield a total score for each of the five factors. Clear support has been found for the psychometric qualities of the BFQ-C in children and adolescents from various countries, such as Iran. Cronbach’s alpha coefficients ranged from 0.82 to 0.95 (Del Barrio et al., 2006; Barbaranelli et al., 2008).

#### Emotional Valences Task

The videos from the Amsterdam Dynamic Facial Expression Set–Bath Intensity Variations (ADFES-BIV; Wingenbach et al., 2016), which is based on the ADFES (van Der Schalk et al., 2011), were used as a task. In this task, there are two aspects of facial expressions; the first, basic emotions such as anger, disgust, fear, happiness, sadness, and surprise. The second aspect involves complex emotions such as contempt, embarrassment, and pride. Each of the emotions is illustrated by 12 encoders: 7 males and 5 females. For each of the 120 videos from the Northern European set (12 encoders × 10 expressions), there are different stages of expression: low, high, and intermediate, yielding 370 videos. The length of each of the videos is 1040 ms, or 26 frames with a frame rate of 25/sec (Wingenbach et al., 2016).

In the present study, the task was presented on a computer monitor (19 “) in the resolution of 1024 × 768, appearing centrally in grayscale against a black background. The task was made in PsychoPy in Python software according to basic emotions (the 7 emotional expressions + neutral; 252 videos). On each trial, a fixation cross was presented in the center of the screen for 500 ms followed by the stimulus presented for 1040 ms. We set 10 min for presenting videos randomly. For answering to the valences of dynamic faces, we designed a five point Likert; 0 to 4 (0 and 1 refer to negative valences, 2 refers to neutral valence, and 3 and 4 refer to positive valences). An infinite response time was chosen to avoid limiting participants in their answer time producing trials with no response. Firstly, we requested participants read the instruction that was presented instruction of five Likert answer shit and how mean of each point of Likert that shows on the monitor below the videos; 7 emotions^∗^12 encoders^∗^3 levels. The emotions consisted of anger, disgust, fear, happiness, sadness, surprise, and neutral face. Secondly, it was requested when participants are ready to click the screen to start the task. The mouse cursor only appeared for the emotion labeling display within trials. The output of the task was put into Excel format. For each face, the mean of valences was analyzed.

#### EEG Recording

Brain activities were recorded by an EEG (Mitsar Co., Ltd., Saint Petersburg, Russia). The device includes 19 main electrodes (Fp1, Fp2, F3, F4, F7, F8, Fz, C3, C4, Cz, P3, P4, Pz, T3, T4, T7, T8, O1, and O2), two reference electrodes (A1 and A2), and a ground electrode (Fpz), according to the 10–20 system of electrode placement. The data were collected using a sampling rate of 250 Hz and filtered in WINEEG software with a frequency band of 1 to 25 Hz with a notch filter of 45–55 Hz. Linked Ear references were used with all EEG. The electrolytic gel was applied and each site gently abraded until impedances were below 10 kΩ. Eyes-closed and eyes-open conditions were used for recording signals that were 3 min each in duration. During the eyes-closed condition, we instructed the participants to place their hands on their knees, half-open their mouths, and avoid blinking or opening the eyes. The eyes-open condition had similar instructions except that we requested them to additionally fixate their eyes on a central point.

After recording the signals, the data were saved in EDF+ format in WINEEG and opened in Neuroguide software. The artifacts were rejected by automatic rejection method. The criteria of automatic rejection included drowsiness, eye-movement, and muscle with a high sensitivity. After that, a 1-s at a 250 sample rate, artifact-free epochs with a Hanning window (50% overlapping) was extracted through Neuroguide software and submitted to the Fast Fourier Transform (FFT; the resolution was 1 Hz). To address the aims of the study, frontal alpha (8–12 Hz) asymmetry indices were calculated by computing asymmetry scores (log [left]–log [right]) for mid-frontal (F3–F4), frontal pole (Fp1–Fp2), and lateral frontal (F7–F8). Positive scores indicate greater alpha power at left compared to right frontal electrode sites, and therefore greater relative right-sided frontal activity. According to reliability of EEG recording, a split test was conducted. The split test showed that the reliability is 0.96 (*SD*: 0.02). The mean of alpha across the scalp in the eyes-closed condition was 64.52 (median: 61.30, *SD*: 31.72). To reduce artifacts in our data, eyes-closed condition was applied for analyzing data.

#### Procedure and Data Analysis

The study was approved by the Ferdowsi University of Mashhad, Department of Psychology Committee which is the University’s body responsible for providing ethics approval (code: 3/44734). The consent obtained from all participants above the age of 16 and from the parents of participants below the age of 16 was both written and informed.

**FIGURE 1:**
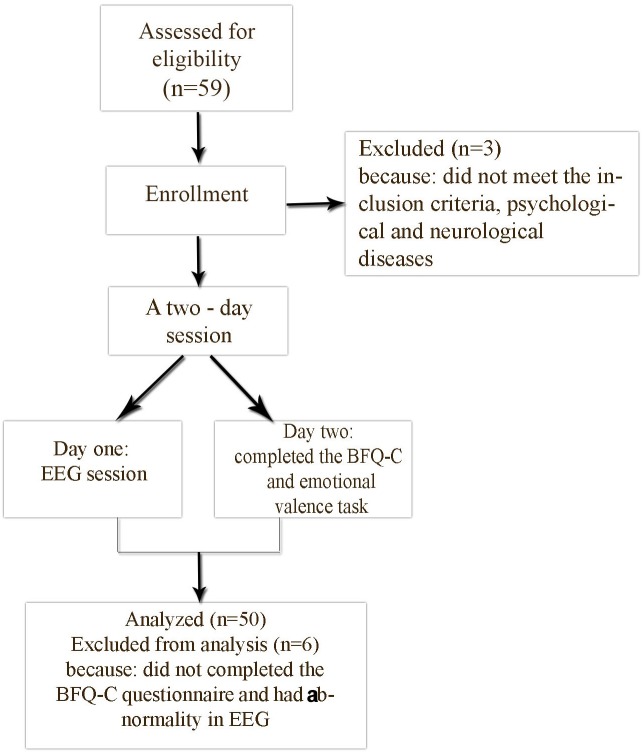
The flowchart of the research’s procedure.

In day one, prior to entering the Polyclinics of Clinical Psychology (QEEG) laboratory, researchers advised participants about the experimental conditions. After that, participants entered the lab. Initial recording was done with eyes open and eyes closed in a relaxed state. In the second day, participants completed the BFQ-C and the emotional valence task. After collecting the information, the data were entered in SPSS 22 (SPSS Inc., Chicago, IL, United States) (see Figure 1). In order to analyze the data, we used a descriptive statistic (arithmetic mean, standard deviation, and Kurtosis) and inferential statistics, including Pearson correlation, and a multiple regression, General Linear Model (GLM)-Univariate.

## Results

Firstly, we chose Kurtosis considering bootstrap method (95% confidence interval) in order to examine statistical power of variables. Kurtosis of neuroticism, fear, joy, neutral, surprise, disgust, anger, sad, frontal pole (Fp1–Fp2), mid-frontal (F3–F4), and lateral frontal (F7–F8) were -0.78, 0.06, 2.41, 5.37, 0.14, 6.99, 1.51, 0.58, 0.33, -0.68, and -0.85, respectively. Considering these results of Kurtosis, variables had a good normality for testing hypotheses.

Table 1 illustrates information about mean and standard deviation (SD) for neuroticism, frontal asymmetry, and emotional valences of faces. Mean (SD) for neuroticism, frontal pole, mid-frontal, and lateral-frontal were 37.24 (9.34), -2.71 (7.37), -6.19 (10.67), and -9.22 (25.58), respectively. As it is seen, participants rated disgust more negative valence than others. Also, participants rated happy more positive valence than others.

**Table 1:** Mean and SD for neuroticism, asymmetry at the frontal pole (Fp1–Fp2), mid-frontal (F3–F4), lateral-frontal (F7–F8), and emotional valences of the face.

Variable	Mean	SD
Neuroticism	37.24	9.34
Fp1–Fp2	-2.710	7.375
F3–F4	-6.197	10.676
F7–F8	-9.222	25.587
Fear	1.371	0.636
Happy	3.194	0.466
Disgust	0.987	0.630
Anger	1.123	0.562
Sad	1.246	0.501
Surprise	1.693	0.630
Neutral	2.014	0.420


### Correlational Findings

To test the correlations between eleven variables, we applied the percentile bootstrap (95% confidence interval, *p*-value 0.05). The bootstrap method is a resampling multiple comparisons procedure that allows assigning measures of accuracy (Efron and Tibshirani, 1994; Westfall, 2011).

According to the Pearson correlation test, the trait of neuroticism was found to be negatively correlated with the fear valence (*p* < 0.01). There was no significant correlation between neuroticism with happiness, anger, and neutral valences (Table 2). There was a significant a significant relationship between neuroticism and disgust (*p* < 0.05). Moreover, there was a significant relationship between neuroticism and sadness (*p* < 0.01). Also, there was a significant relationship between neuroticism and surprise (*p* < 0.05).

**Table 2:** Correlation between neuroticism, emotional valences of the face, asymmetry at the frontal pole (Fp1–Fp2), mid-frontal (F3–F4), and lateral-frontal (F7–F8).

Variables	Neuroticism		Fp1–Fp2		F3–F4		F7–F8	
	*r*	Lower (upper)	*r*	Lower (upper)	*r*	Lower (upper)	*r*	Lower (upper)
Neuroticism	1	1	0.039	-0.188 (0.374)	0.356*	0.082 (0.573)	0.273*	-0.51 (0.669)
Fear	-0.425**	-0.653 (-0.177)	-0.307*	-0.519 (-0.003)	-0.669**	-0.782 (-0.496)	-0.393**	-0.694 (-0.153)
Happy	-0.245	-0.576 (0.115)	0.106	-0.131 (0.352)	-0.157	-0.478 (0.128)	0.074	-0.276 (0.225)
Disgust	-0.320*	-0.536 (-0.140)	-0.090	-0.101 (0.345)	-0.069	-0.313 (0.204)	-0.153	-0.380 (0.167)
Anger	0.151	-0.103 (0.402)	0.164	-0.158 (0.427)	0.159	-0.135 (0.513)	0.032	-0.244 (0.392)
Sad	-0.381**	-0.600 (-0.145)	0.029	-0.341 (0.266)	-0.150	0.525 (0.157)	-0.163	-0.446 (0.191)
Surprise	-0.341*	-0.657 (0.001)	0.073	-0.098 (0.296)	-0.103	-0.446 (0.246)	-0.046	-0.427 (0.219)
Neutral	0.029	-0.289 (0.200)	0.055	-0.212 (0.448)	0.030	-0.347 (0.220)	0.073	-0.249 (0.345)


*^∗∗^*p < 0.01* and ^∗^p < 0.05*.

In terms of neuroticism and EEG asymmetry, positive and significant relationships existed between neuroticism, F3–F4, and F7–F8 (*p* < 0.05). With respect to the correlation between emotional valences and EEG asymmetry, results showed significant relationships between fear, Fp1–Fp2, F3–F4 and F7–F8 (*p* < 0.01) that the magnitude of correlation is more on the relationship between F3–F4 and the valence of fear. However, there was no significant relationship between other emotional valences and EEG asymmetry. Regarding the bootstrap method, the correlational results were in 95% confidence interval. Thus, based on the sample size of present study, the results can be statistically generalized to the population.

### General Linear Model Analysis

Evaluation of different variables revealed significant relationships between neuroticism, the valence of fear and frontal asymmetry (Table 2). To test the predicted the valence of fear by interaction for the F3–F4 asymmetry and neuroticism, a multiple regression of activity was conducted using SPSS the General Linear Model (GLM)-Univariate procedure. The dependent variable was the valence of fear as a continuous variable, and the independent variable was neuroticism and the moderator variable was the F3–F4 asymmetry that were continuous variables. Before conducting GLM, we checked the multiple regression assumptions. To test the assumption of variables normality, Shapiro-Wilk was applied. The results showed that the assumption of normality is met. Additionally, variables had no significant outliers.

We set a custom model considering a main effect of neuroticism, and a two-way interaction of neuroticism^∗^F3–F4 asymmetry. The significant level was 0.05 with 95% confidence interval. Also, the effect size was estimated by eta-squared. All procedure was applied considering the percentile bootstrap (95% confidence interval).

Table 3 illustrates the GLM results. The valence of fear was significantly influenced by neuroticism (*F* = 16.55, *P* = 0.0005, eta^2^ = 0.26). The effect size of this result was large and the observed power was strong. Moreover, frontal asymmetry could significantly moderate in the relationship between neuroticism and the valence of fear (*F* = 27.90, *P* = 0.0005, eta^2^ = 0.372). According to the interaction analysis, the effect size of this result was large and the observed power was high.

**Table 3:** Summary of interactions between neuroticism, frontal asymmetry to predict the valence of fear.

Source	Mean square	*Df*	*F*	Sig	Eta^2^	Observed power
Neuroticism	3.597	1	16.555	0.0005	0.260	0.979
Neuroticism^∗^frontal asymmetry	6.063	1	27.900	0.0005	0.372	0.999


*^∗^p < 0.05*.

## Discussion

Based on the model of brain activity, facial expressions, and personality traits presented in our study, it can be hypothesized that emotional valences may be predicted by neuroticism and frontal asymmetry in the frontal region. In order to examine this hypothesis, Pearson correlations and the GLM-univariate were applied. The results showed that neuroticism correlates with the valences of fear, disgust, sadness, and surprise, but not with happiness, anger, and neutral faces. Moreover, it was found that N was significantly positively correlated with mid-frontal asymmetry (F3–F4), whereas no correlation was found between N and frontal pole (Fp1–Fp2). There was also a correlation between N and lateral-frontal (F7–F8). We found significant negative correlations between the valence of fear, Fp1–Fp2, F3–F4, and F7–F8, while there was no significant correlation between the other valence of facial emotional expressions and frontal asymmetry. The interaction findings revealed that a significant main effect of neuroticism on the valence of fear was found. Furthermore, the results showed that frontal asymmetry could significantly modulate the relationship between neuroticism and the valence of fear.

We firstly examined the relationships between, emotional valences and neuroticism. The results showed that N is negatively related to valences of fear, disgust, sadness, and surprise. In agreement with our results, previous studies have found relationships between neuroticism and negative emotions (Stewart et al., 2005; Haas et al., 2008; Trnka et al., 2012). For instance, Haas et al. (2008) have shown that the sustained processing of negative stimuli associated with neuroticism is specific to sad facial expressions, not happy or fearful facial expressions. Nonetheless, our behavioral results partially supported Haas et al. (2008) findings. They did not find any association between N and the fearful expression. Trnka et al. (2012) showed that people who score high for N scores negatively emotional valences and assesses emotions as a more unpleasant. They also showed that there are significant negative relationships between neuroticism, disgust, and other negative emotions. This finding suggests that individuals with the N trait tend to evaluate positive emotional faces negatively.

As it was observed that there is a significant negative relationship between neuroticism and the valence of sad faces. It is inferred that people who scores high for N factor negatively evaluate sad faces. Previous studies have shown that neuroticism is related to depression (Krueger et al., 1996; Xia et al., 2011; De Moor et al., 2015; Navrady et al., 2017). For instance, Navrady et al. (2017) revealed that neuroticism may be a risk factor increasing depression. It has been shown that N factor includes a tendency to be worried and anxious, and N is related to the experience of negative emotion. In fact, subjects with a high trait N are more distressed by negative moods and tend to be more psychologically reactive to stressors (Reynaud et al., 2012). Finally, this finding supported Eysenck model (Eysenck and Eysenck, 1985) and the big five model of personality traits (McCrae and Costa, 1999).

As we calculated the alpha wave in the eyes-closed condition, we observed a negative relationship between the valence of the fearful face and frontal asymmetry. It is believed that negative ratings to fearful faces demands greater alpha activity in the left frontal region. In the other word, when the alpha band activates in an area during eyes-closed condition, the cortical arousal decreases in that area (Lindgren et al., 1999). Furthermore, N factor is positively related to the frontal asymmetry activity in the eyes-closed condition. It is assumed that increasing of the level of neuroticism enhances the level of frontal asymmetry activity in the alpha band. Our data revealed that the left frontal arousal level is lower than the right frontal region. As several studies have shown that cortical arousal is related to decreasing of alpha activity (Barry et al., 2007, 2009), it seems that decreasing of alpha activity in the right frontal region is associated with increased cortical arousal during eyes-closed condition, which may related to the valence of fear and neuroticism.

These findings are consistent with previous studies (Gale et al., 2001; Compton and Weissman, 2002; Coan and Allen, 2003, 2004; Minnix and Kline, 2004; Blackhart et al., 2006; Pavlenko et al., 2009; Harmon-Jones et al., 2010). In terms of the relationship between neuroticism and the frontal asymmetry, Minnix and Kline (2004) revealed that neuroticism is inversely correlated to the left mid-frontal activity. According to the relationship between the frontal asymmetry and the valence of facial processing, Coan and Allen (2003) suggested that EEG asymmetries were characterized by higher left-side activity for positive emotions, and greater right-side activity for negative emotions. Moreover, Blackhart et al. (2006) showed that greater relative left frontal EEG activity is related to positive, approach-related emotions, whereas greater relative right frontal EEG activity is related to negative, withdrawal-related emotions. In fact, in terms of valence of emotions, our findings revealed that EEG frontal asymmetry in resting state is just related to valence ratings of fearful face. Though previous studies have suggested that EEG frontal asymmetry is associated with emotions, we could not completely support these studies. As a result of classification of emotions in positive and negative in previous investigations, we classified dynamic faces in specific types, including fear, disgust, angry, happy, sad, surprise, and neutral.

The relationships findings indicated valence ratings of fearful face is related to frontal asymmetry and neuroticism, whereas there is no relationship between frontal asymmetry and other emotions. Though neuroticism was related to other emotions except happy, anger, and neutral. It is a question; why did occur this relationship between neuroticism and emotions, and did not for frontal asymmetry and other emotions (except fear)? Our findings may answer a novel question. We should have several hypotheses to answer the question.

First, we believe that though frontal asymmetry and neuroticism are clarified as traits and individual differences, neuroticism is a personality factor that is a stable pattern of behaviors, thoughts, emotions, and motivations (McCrae and Costa, 1999). While, in the literature, primary models of frontal asymmetry indicated it is an emotional processing, whereas new models, Coan et al. (2006) and Grimshaw and Carmel (2014), proposed it is a cognitive processing. As a result, it may be there are limited features in frontal asymmetry than in neuroticism. Nonetheless, there were magnitude correlations between neuroticism and frontal asymmetry. As a limitation, we investigated total score of neuroticism, we recommend that it will be novel if future investigators examine subscales of neuroticism to test a relationship between subscales of neuroticism and frontal asymmetry.

Secondly, our research has done in adolescence that anxiety disorder is most commonly psychological disorder in this stage (Grant, 2013). Studies have shown that a relationship between attentional bias toward fear threating and anxiety is highlighted (Dudeney et al., 2015). Therefore, it seems that adolescence demands more cognitive control toward fearful expressions to encounter with fearful stimulus. In the other word, it is probably that frontal asymmetry has a cognitive mechanism. Thus, it is inferred that the activity of frontal is more in the right-side than in the left-side that low activity in the left frontal region expresses a low inhibition of fear. The asymmetric inhibition model (Grimshaw and Carmel, 2014) indicates left dlPFC is hypothesized to inhibit negative information and right dlPFC is hypothesized to inhibit positive information. However, it needs to examine cognitive mechanisms of emotions and frontal asymmetry in adolescence.

Thirdly, previous studies have shown that dlPFC is a top-down process to regulate the amygdala (Sagliano et al., 2016). dlPFC is related to threats, including external (fear) and social (anger) threats (De Raedt et al., 2010; Sylvester et al., 2017). However, our findings indicated that frontal asymmetry is associated with fear. Balderston et al. (2017) showed that dlPFC plays a key role in effect of anxiety that this study is consistent with our hypothesis. Though dlPFC regulates the amygdala in processing threats, we assume that frontal asymmetry may play a role in regulating external threats. Thus, it is possible that there are another contributions in other regions such as the parietal asymmetry to regulate the amygdala processing. To support, Grimshaw et al. (2014) showed that the fronto-parietal network controls attention to threats. Therefore, we recommend that the fronto-parietal network asymmetry should be examined to predict social threats (angry faces).

According to the last aim, we presented a hypothesis to test interactions of neuroticism and mid-frontal asymmetry to predict valence ratings of fearful faces. GLM results showed that EEG mid-frontal asymmetry can strongly moderate the relationship between neuroticism and the valence of fear. Coan and Allen (2004) proposed a moderator role of frontal asymmetry to predict emotional responses. Therefore, neuroticism would predict that individuals who show greater right frontal activity at resting-state are at higher negative valences for fearful faces. As a result, the interactions of neuroticism and frontal asymmetry may be a psychopathological risk in adolescence for anxiety disorders in the future.

As a strength of present study, we applied the bootstrapping method for the multiple comparisons. Moreover, we compared the classical and new models of frontal asymmetry that can cause novel answers and questions in this area of study. There were obvious limitations to our approach. First and foremost, this is a correlational study between variables and can not be used to establish a causal relationship between asymmetries, emotional processing and personality. Second, the sample size was small and it is recommended that future studies should investigate the hypotheses in a large sample and in different gender. Third, we investigated total score of neuroticism, we recommend future investigators examine subscales of neuroticism to test a relationship between subscales of neuroticism and frontal asymmetry. Moreover, to extend our knowledge in this area, we recommend that researchers should use other neuroscience methods such as fMRI or LORETA to find subcortical aspects of fronto-parietal network in order to answer the question: what is the mechanism of frontal asymmetry? Does it have a cognitive function or emotion function? Thus, it will be helpful for scientists in this field.

In conclusion, the finding of this study suggested that in adolescence, similar to adulthood, neuroticism is correlated with the valence of fear, angry, disgust, and surprise. Furthermore, the results showed that frontal asymmetry is related to neuroticism and the valence of fear. Our findings presented a hypothesis in cognitive mechanisms of frontal asymmetry for further investigations. Also, the findings showed a significant interaction between neuroticism and mid-frontal asymmetry to predict the valence of fear. Therefore, neuroticism trait and mid-frontal EEG asymmetry may serve as a risk indicator for psychopathology.

## Ethics Statement

Parents of participants under 16 years old and participants above 16 years old completed the consent form. All procedures performed in studies involving human participants. The study was approved by the Ferdowsi University of Mashhad, Department of Psychology Committee which is the University’s body responsible for providing ethics approval (Code: 3/44734).

## Author Contributions

All authors listed have made a substantial, direct and intellectual contribution to the work, and approved it for publication.

## Conflict of Interest Statement

The authors declare that the research was conducted in the absence of any commercial or financial relationships that could be construed as a potential conflict of interest.
